# A Case Series on the Effectiveness of Abdominal Belts for Postural Blood Pressure Disorders in Parkinson’s Disease

**DOI:** 10.7759/cureus.86109

**Published:** 2025-06-16

**Authors:** Katsunori Yokoi, Keisuke Suzuki, Akiko Yamaoka, Masahisa Katsuno, Yutaka Arahata

**Affiliations:** 1 Department of Neurology, National Center for Geriatrics and Gerontology, Obu, JPN; 2 Department of Neurology, Nagoya University Graduate School of Medicine, Nagoya, JPN

**Keywords:** 24-hour ambulatory blood pressure monitoring, abdominal belt, case series, orthostatic hypotension, parkinson’s disease

## Abstract

Orthostatic hypotension (OH) is a common non-motor complication of Parkinson’s disease (PD), significantly affecting the quality of life and increasing fall risk. While pharmacological treatments are available, their efficacy is often limited. Abdominal belts have been proposed as a non-pharmacological intervention, but their effects on blood pressure (BP) fluctuations remain unclear. In this case series, we examined four patients with PD and OH, all of whom underwent 24-hour ambulatory BP monitoring (ABPM) before and after using an abdominal belt. The belt was applied with a pressure of 20 ± 2 mmHg and worn during waking hours. The mean systolic BP (SBP) and diastolic BP (DBP) during daytime and nighttime were compared using the Mann-Whitney U test. BP variability was assessed. Three patients demonstrated improved BP stability with abdominal belt use, particularly in reducing postprandial hypotension. One patient (Case 1) discontinued use due to discomfort and showed no significant BP changes. In Case 3, SBP increased from 117.85 ± 5.92 mmHg to 162.74 ± 10.80 mmHg (p = 0.002), and DBP from 66.35 ± 3.36 mmHg to 91.19 ± 5.24 mmHg (p = 0.001). Case 4 also exhibited a significant increase in SBP (p = 0.005). However, BP fluctuations persisted, and nocturnal BP remained unchanged. We concluded that the use of an abdominal belt may help stabilize BP in patients with PD and OH during daytime activities, but does not eliminate BP variability. Studies with more patients are needed to confirm these findings and refine recommendations for abdominal belt use in PD management.

## Introduction

Parkinson’s disease (PD) is the second most common neurodegenerative disorder after Alzheimer’s disease, with prevalence increasing with age. It is primarily caused by the progressive degeneration of dopaminergic neurons in the substantia nigra, leading to both motor and non-motor symptoms. Epidemiological studies report a PD prevalence of 3.1% in individuals aged >75 years and 4.5% in those aged >85 years [1,2]. Among the non-motor symptoms, orthostatic hypotension (OH) is a frequent and clinically significant complication that severely impacts daily functioning.

OH is clinically defined as a sustained reduction of at least 20 mmHg in systolic blood pressure (SBP) or 10 mmHg in diastolic blood pressure (DBP) within three minutes of standing up or head-up tilt to ≥60 degrees [3]. In PD, OH is primarily attributed to autonomic dysfunction caused by degeneration of the peripheral sympathetic nervous system and central autonomic pathways [4]. This autonomic impairment leads to inadequate vasoconstriction upon standing, resulting in insufficient venous return, decreased cardiac output, and subsequent BP drops. OH not only contributes to physical disability and reduced quality of life but also increases the risk of falls by 2.6 times, potentially affecting prognosis [4,5]. Furthermore, OH in PD has been associated with cognitive decline and increased mortality risk, underscoring the need for effective management strategies [6,7].

The current treatment options for OH in PD primarily include pharmacological interventions, such as midodrine, an alpha1-adrenergic receptor agonist; droxidopa, a precursor of norepinephrine; and fludrocortisone acetate. However, these treatments often have limited efficacy owing to side effects such as supine hypertension, short duration of action requiring frequent dosing, and variability in individual responses, leading to inconsistent therapeutic outcomes [8,9]. Consequently, non-pharmacological approaches, including lifestyle modifications and mechanical interventions, such as elastic stockings and abdominal belts, have been explored as complementary strategies for managing OH [10]. Abdominal binders can help mitigate OH by increasing venous return and stabilizing blood pressure (BP) [11]. Despite their theoretical benefits on orthostatic tolerance, evidence supporting their effectiveness remains limited. Current clinical guidelines for PD do not universally recommend their use, reflecting a lack of consensus on their efficacy and the need for further research [3].

BP variability (BPV) is a clinically significant factor in patients with PD and OH, as it has been linked to an increased risk of cardiovascular events, cognitive decline, and disease progression [12,13]. Monitoring BPV is essential for assessing the effectiveness of OH interventions, and 24-hour ambulatory BP monitoring (ABPM) has been identified as a useful tool for evaluating these fluctuations [11]. However, comprehensive studies on the impact of abdominal binders on BPV in patients with PD remain limited [3].

To address this gap, we conducted a case series of four patients with PD and OH who underwent 24-hour ABPM before and after the application of an abdominal belt. We aimed to provide insights into the effectiveness of abdominal belts in stabilizing BP and reducing variability throughout the day. Ultimately, our findings may inform clinical guidelines and improve treatment strategies for OH in PD, offering a more personalized approach to non-pharmacological management [11,13].

## Case presentation

Study design and ethical approval

We present a case series of four patients with OH associated with PD who used abdominal belts. Changes in BP were assessed using a 24-hour BP monitor by comparing measurements taken with and without the abdominal belt for each patient. This study was approved by the Ethics Committee of the National Center for Geriatrics and Gerontology (NCGG) (approval number: 1450-3). Since the information was extracted in a manner that prevents individual identification through the opt-out process, individual informed consent was not required. However, for the four patients included in this study, verbal and written consent was obtained for the use of lidocaine injections and muscle hardness gauges before administration. Verbal informed consent was obtained for the use of patient information in research.

Inclusion criteria

To be included in this study, patients needed a diagnosis of PD, a diagnosis of OH, and daily life-impairing BP fluctuations. Moreover, they had to be willing to undergo 24-hour BP monitoring and use an abdominal belt. OH was defined as a decrease in systolic BP (SBP) of ≥20 mmHg and a decrease in diastolic BP (DBP) of ≥10 mmHg within three minutes of transitioning from a supine to a standing position [14]. This threshold (≥20 mmHg in SBP) was also used as the criterion for interpreting clinical changes in response to the intervention.

Exclusion criteria

Patients were excluded if they had OH due to a condition other than PD, cognitive decline preventing the ability to follow instructions, inability to wear an abdominal belt for any reason, or were unwilling to wear a 24-hour BP monitor.

Procedure

**Figure 1:**
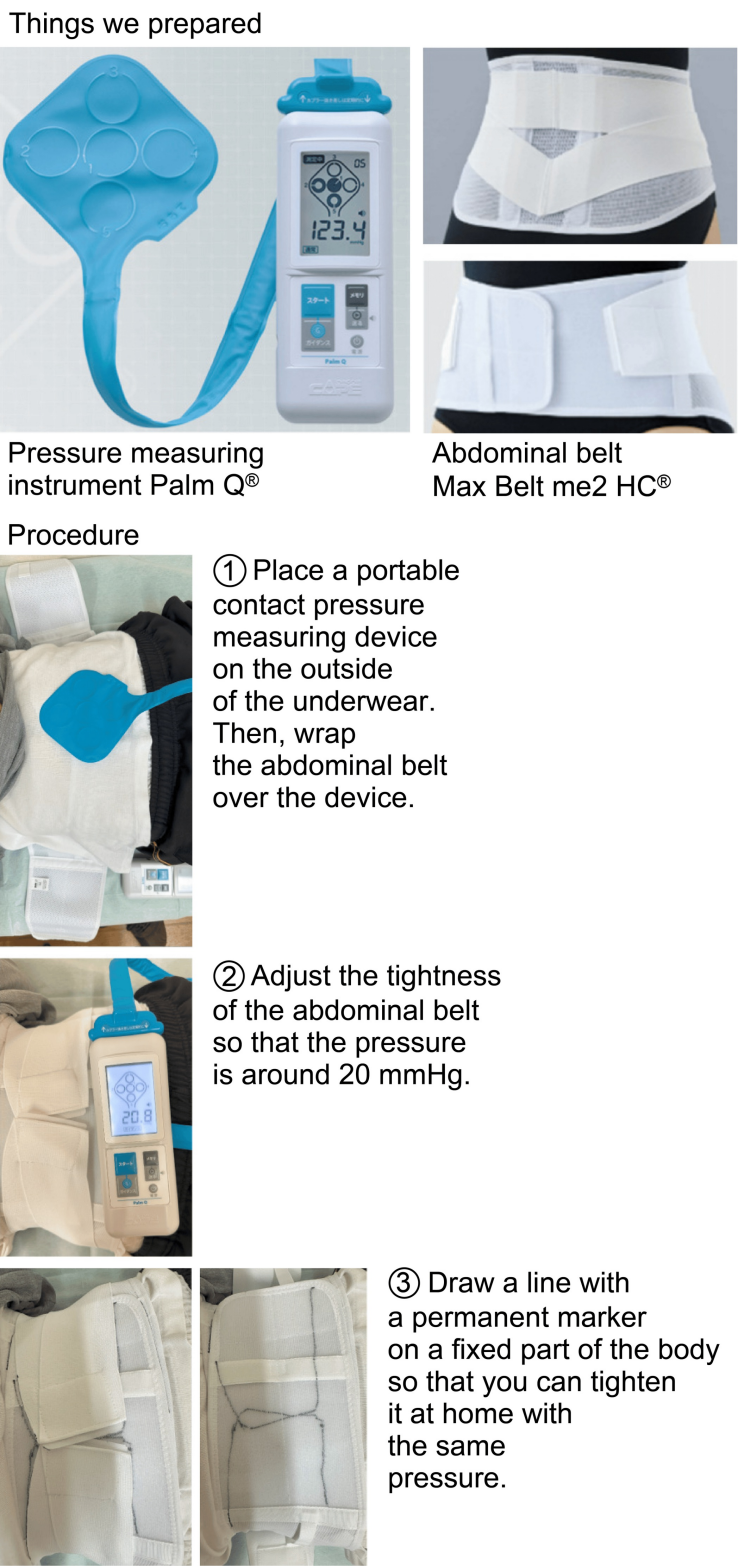
Protocol for using the abdominal belt Permission to use the product names and images of Palm Q® (CAPE Co., Ltd.) and Max Belt me2 HC® (Nippon Sigmax Co., Ltd.) was obtained from the respective manufacturers on April 24, 2025.

First, baseline BP was measured using a 24-hour BP monitor before abdominal belt use. Then, patients were fitted with the abdominal belt and provided with instructions on its use (Figure 1). On the following day, patients wore the abdominal belt, and BP was measured again using the 24-hour BP monitor. Patients were instructed to wear the abdominal belt from 07:00 to 22:00 during daytime activities. Although actual adherence was based on patient self-report, all participants indicated general compliance with this schedule. Finally, changes in BP before and after using the abdominal belt were compared.

The abdominal belt was applied to exert an abdominal wall pressure of 20 ± 2 mmHg [15]. Markings were made to ensure consistent tightening pressure. The pressure measurement device used was the PalmQ® (CAPE Co., Ltd., Yokosuka, Kanagawa, Japan), and the abdominal belt was the Max Belt me2HC® (Sigmax Co., Ltd., Tokyo, Japan).

BP was continuously monitored while wearing the abdominal belt, including during sleep. All four patients were monitored for 24 hours on the day following their initial use of the abdominal belt. One patient (Case 1) refused to wear the abdominal belt during 24-h monitoring; hence, measurements were taken without it. This case is included as an exception for comparison purposes.

For all patients, medication was not altered for at least one month prior to the use of the abdominal belt and remained unchanged until 24-hour BP monitoring was completed to accurately assess the device’s effectiveness.

BP measurements were categorized into two periods: daytime (07:00-22:00, excluding bedtime) and nighttime (23:00-06:00, during sleep).

To evaluate the impact of abdominal band application on postprandial blood pressure regulation, we compared the maximum systolic and diastolic blood pressure during the pre-lunch period (12:00-13:00) with the minimum values during the post-lunch period (13:00-14:00), both before and after the intervention, across all four cases.

BP values with and without the abdominal belt were compared. Changes in BP for each patient were analyzed using the Mann-Whitney U test. Additionally, standard deviation and coefficient of variation were calculated and evaluated. All statistical analyses were conducted using EZR, a software package for R that enhances standard R commands by incorporating statistical functions commonly used in biostatistics [16].

Case 1

Background

A 78-year-old man initially presented with OH and was subsequently diagnosed with PD. Despite adjustments in his regimen, including L-Dopa, midodrine (8 mg), droxidopa (600 mg), and fludrocortisone (0.05 mg), the patient continued to experience frequent episodes of loss of consciousness and difficulty standing (Table 1, Case 1).

**Table 1:** Demographic and clinical characteristics of four patients with Parkinson’s disease and orthostatic hypotension. Characteristics included age, sex, disease duration, Parkinson’s disease phenotype, levodopa equivalent daily dose (LEDD), dopaminergic agents, and use of pressor or antihypertensive medications. All medications were maintained without change for at least one month before the intervention.

	Age	Sex	Years from onset	Parkinson’s disease phenotype	Levodopa equivalent daily dose (LEDD)	Specific dopaminergic drugs (/day)	Pressor agents (/day)	Anti-hypertensive drugs (/day)
Case 1	78	Male	5	Postural instability and gait difficulty type	675 mg/day	Levodopa/benserazide 600 mg, zonisamide 25 mg, safinamide 50 mg	Droxidopa 600 mg, midodrine 4 mg	Antihypertensive discontinued at onset
Case 2	81	Male	7	Akinetic-rigid type	569 mg/day	Levodopa/carbidopa/entacapone 300 mg, levodopa/benserazide 50 mg, zonisamide 50 mg, safinamide 50 mg, istradefylline 20 mg	Midodrine 6 mg	Arotinolol 10 mg
Case 3	83	Male	4	Postural instability and gait difficulty type	450 mg/day	Levodopa/benserazide 450 mg	Droxidopa 600 mg, midodrine 8 mg, fludrocortisone 0.05 mg	No history of antihypertensive medication use
Case 4	74	Female	9	Postural instability and gait difficulty type	766 mg/day	Levodopa plus benserazide 350 mg, rasagiline 1 mg, opicapone 25 mg, amantadine 200 mg	Droxidopa 600 mg	No history of antihypertensive medication use

Intervention

The patient was advised to use an abdominal belt; however, he discontinued its use due to difficulties managing urination while wearing it. Although the patient did not report discomfort, pressure, or breathing difficulties while wearing the belt, he found it challenging to lower his underwear, leading to discontinuation the following day.

BP Changes

**Figure 2:**
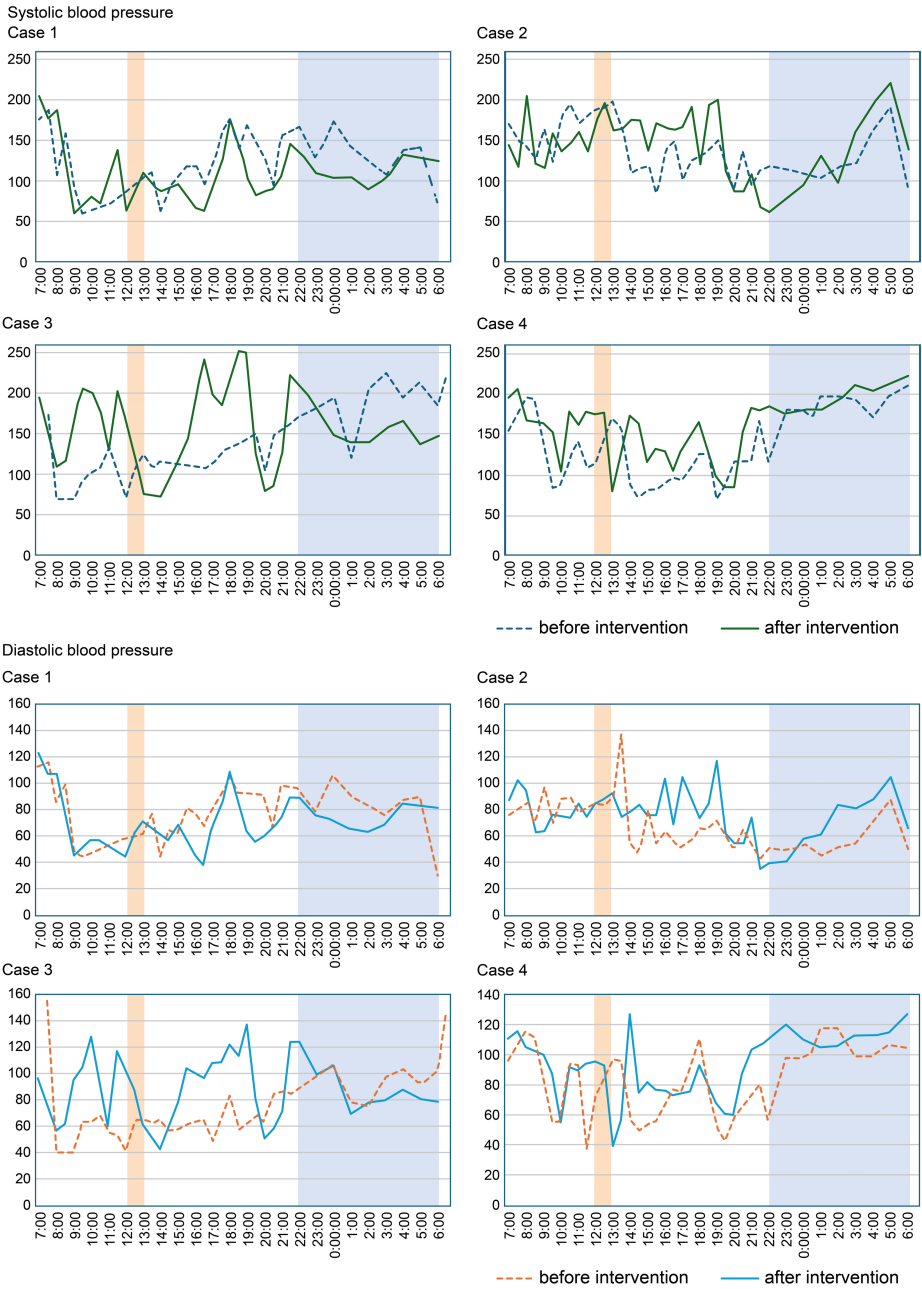
Changes in 24-hour blood pressure before and after using the abdominal belt Colored areas indicate the periods when the abdominal belt is removed and when the patient sleeps at night.

Twenty-four-hour BP monitoring without the abdominal belt revealed minimal BP changes, with no significant differences observed between daytime and nighttime values (Figure 2, Case 1, Table 1). The change in blood pressure before and after lunch was modest, with a systolic blood pressure (SBP) drop of 23 mmHg before the intervention and 27 mmHg after. Diastolic blood pressure (DBP) changes were also small (5 mmHg before, 9 mmHg after). No clear trend was observed in response to the intervention (Table 2).

**Table 2:** Changes in blood pressure before and after lunch Maximum systolic and diastolic blood pressures were extracted from the pre-lunch period (12:00–13:00), and minimum values from the post-lunch period (13:00–14:00). ΔSBP and ΔDBP were calculated as the difference between pre-lunch maximum and post-lunch minimum for each parameter.

			Pre-lunch Maximum SBP	Post-lunch Minimum SBP	ΔSBP or DBP (Drop)
Case 1	Before	Systolic	106	63	43
		Diastolic	62	45	17
	After	Systolic	111	88	23
		Diastolic	71	57	14
Case 2	Before	Systolic	200	109	91
		Diastolic	89	53	36
	After	Systolic	197	163	34
		Diastolic	88	75	13
Case 3	Before	Systolic	124	107	17
		Diastolic	65	62	3
	After	Systolic	128	73	55
		Diastolic	87	43	44
Case 4	Before	Systolic	172	73	99
		Diastolic	97	50	47
	After	Systolic	177	80	97
		Diastolic	93	39	54

Case-Specific Discussion

This case highlights that practical issues, such as urination difficulties, can limit the sustained use of abdominal belts for OH management in patients with PD, even in the absence of discomfort.

Case 2

Background

An 81-year-old man was diagnosed with PD and dementia. The disease initially manifested with left-sided muscle stiffness, followed by cognitive decline, hallucinations, daytime somnolence, and hypotension. Despite treatment with L-Dopa and midodrine (6 mg), hypotension and episodes of loss of consciousness persisted (Table 1, Case 2).

Intervention

The use of the abdominal belt led to an improvement in daytime consciousness. The patient reported no discomfort, pressure, or breathing difficulties, and the belt was well tolerated.

BP Changes

Although statistically significant BP changes were not observed, there was a tendency for both SBP and DBP to increase during daytime periods with belt use. BP fluctuations persisted throughout monitoring (Figure 2, Case 2, Table 1). The change in blood pressure before and after lunch was pronounced, with a pre-intervention SBP drop of 91 mmHg and DBP drop of 25 mmHg, indicating clear postprandial hypotension (PPH). These drops were substantially reduced after the intervention (ΔSBP = 34 mmHg, ΔDBP = 5 mmHg), suggesting a notable stabilizing effect of the abdominal band (Table 2).

Case-Specific Discussion

Despite ongoing BP variability, the subjective improvement in daytime consciousness suggests that abdominal belt use may offer clinical benefits beyond simple BP elevation.

Case 3

Background

An 83-year-old man diagnosed with PD developed Parkinsonian symptoms four years earlier and was recently diagnosed with OH and daytime somnolence. His treatment included L-Dopa, midodrine (4 mg), and droxidopa (600 mg), but he frequently lost consciousness during meals (Table 1, Case 3).

Intervention 

An abdominal belt was introduced, reducing but not completely eliminating episodes of loss of consciousness. While the patient reported no discomfort or breathing difficulties, the time required to apply the belt limited its use to times when he left his room.

BP Changes

Daytime SBP and DBP significantly increased during belt use. Nighttime SBP significantly decreased after the belt was removed before bedtime (Figure 2, Case 3; Table 1). The change in blood pressure before and after lunch was mild, with no difference in SBP drop between the pre- and post-intervention periods (14 mmHg). DBP changes were also small (10 mmHg before, 6 mmHg after), and no clear intervention effect was observed (Table 2).

Case-Specific Discussion

This case demonstrates that abdominal belt use can effectively stabilize daytime BP and reduce orthostatic symptoms, although practical barriers to frequent use remain.

Case 4

Background

A 74-year-old woman with a nine-year history of PD developed a shuffling gait and later OH secondary to BP fluctuations. Despite treatment with L-Dopa and droxidopa (600 mg), she continued to experience severe dizziness upon standing (Table 1, Case 4).

Intervention

Introduction of an abdominal belt improved symptoms. The patient tolerated the belt well, reporting no discomfort, pressure, or breathing difficulties.

BP Changes

Daytime SBP increased during belt use, and nighttime DBP tended to decrease after belt removal. Although no statistically significant differences were observed, BP values appeared more stable during daytime periods (Figure 2, Case 4; Table 3). The change in blood pressure before and after lunch was inconsistent. SBP dropped by 34 mmHg before the intervention, but the drop increased to 43 mmHg after. DBP also showed a greater decrease post-intervention (from 20 mmHg to 25 mmHg), suggesting no benefit from the abdominal band (Table 2).

**Table 3:** Changes in blood pressure before and after using the abdominal belt. Note: Each value represents the mean blood pressure during daytime waking hours (when the abdominal belt is used). Data are presented as means ± standard errors (SE). SD, standard deviation; CV, coefficient of variation.

			Before			After			U-value	Z-value	p-value	Effect size, r
			Blood pressure	SD	CV	Blood pressure	SD	CV				
Case 1	During activities	Systolic	126.08 ± 7.48	36.64	0.29	110.37 ± 7.79	39.71	0.36	432.5	1.740	0.083	0.241
		Diastolic	80.4 ± 3.92	20.04	0.25	70.26 ± 3.19	21.59	0.31	436.5	1.813	0.071	0.251
	Bedtime	Systolic	128.86 ± 7.77	29.75	0.23	109.86 ± 6.25	13.2	0.12	38	1.725	0.097	0.461
		Diastolic	79.86 ± 8.92	21.85	0.27	73.00 ± 3.08	7.54	0.1	37.5	1.661	0.109	0.444
Case 2	During activities	Systolic	141.14 ± 6.04	31.94	0.23	146.39 ± 6.93	37.94	0.26	399	-0.747	0.459	-0.096
		Diastolic	71.07 ± 4.02	19.5	0.27	77.81 ± 3.52	17.82	0.23	338.5	-1.642	0.102	-0.212
	Bedtime	Systolic	127.25 ± 6.32	30.9	0.24	140.50 ± 14.80	46.84	0.33	28	-0.420	0.721	-0.105
		Diastolic	58.00 ± 4.98	13.17	0.23	73.13 ± 7.15	18.92	0.26	16	-1.680	0.105	-0.420
Case 3	During activities	Systolic	117.85 ± 5.92	29.59	0.25	162.74 ± 10.80	55.06	0.34	178	-3.078	0.002	-0.423
		Diastolic	66.35 ± 3.36	22.73	0.34	91.19 ± 5.24	26.33	0.29	169.5	-3.229	0.001	-0.444
	Bedtime	Systolic	190.13 ± 10.23	29.5	0.16	158.56 ± 11.44	14.39	0.09	55.5	2.468	0.015	0.617
		Diastolic	94.13 ± 4.16	11.02	0.12	85.00 ± 4.28	11.34	0.13	42.5	1.103	0.293	0.276
Case 4	During activities	Systolic	120.07 ± 6.91	35.22	0.29	148.79 ± 6.68	35.36	0.24	221	-2.796	0.005	-0.374
		Diastolic	75.00 ± 3.99	21.87	0.29	85.66 ± 3.92	20.14	0.24	286	-1.730	0.085	-0.231
	Bedtime	Systolic	189.56 ± 9.36	12.96	0.07	197.86 ± 4.02	16.51	0.08	24.5	-1.107	0.288	-0.268
		Diastolic	104.67 ± 2.54	7.2	0.07	113.63 ± 2.56	6.78	0.06	14.5	-2.069	0.043	-0.502

Case-Specific Discussion

This case suggests that consistent use of an abdominal belt can alleviate OH symptoms without discomfort, particularly in patients who are able to tolerate continuous use.

## Discussion

OH is a well-recognized non-motor feature of PD, known to impair quality of life and increase the risks of falls, cognitive dysfunction, and mortality.

The results of this study suggest that in Case 1, where the abdominal belt was not used, there was minimal improvement in symptoms. Although some changes were observed in the coefficient of variation, there was little change in the average BP or daily BP fluctuations over time. Although some degree of blood pressure fluctuation was noted, the overall pattern remained similar before and after the intervention. This case may reflect a relatively stable postprandial hemodynamic response, with limited susceptibility to PPH or limited responsiveness to the abdominal band.

In Case 2, the post-midday BP drop was attenuated, which corresponded with reported symptom improvement. However, there was minimal change in the coefficient of variation or average BP. Notably, this patient was underweight, which may be a distinguishing factor from the others. This case demonstrated the most prominent PPH and a substantial reduction in post-lunch BP drop following the intervention. The abdominal band appeared to be highly effective, suggesting that patients with strong autonomic responses or higher vulnerability may particularly benefit from this intervention.

In Case 3, BP tended to increase significantly with the use of the abdominal belt. Both upper and lower ranges increased, and the mean values of SBP and DBP during the day showed a significant increase. However, there was no significant change in the coefficient of variation. Overall BP variability was minimal, and no apparent effect of the intervention was noted. The PPH itself was mild, potentially limiting the detectability of any treatment effect.

In Case 4, SBP increased overall during the day when the abdominal belt was used, with a significant increase in mean values. Similar to Case 3, there was no significant change in the coefficient of variation. A paradoxical increase in BP drop was observed after the intervention. This may be attributable to intrinsic factors such as autonomic dysfunction, posture-related changes, or activity levels. Additional supportive strategies may be necessary for such cases.

Our analysis of pre- and postprandial blood pressure revealed a substantial reduction in ΔSBP and ΔDBP in Case 2 after abdominal belt use, indicating a possible mitigating effect on PPH. However, only two of the four cases demonstrated statistically significant improvements in daytime BP, highlighting the need to distinguish statistical significance from clinical relevance when interpreting these findings. Although this trend was not observed uniformly across all cases, the data suggest that abdominal compression may benefit selected patients with pronounced PPH patterns. This finding is clinically relevant given the high prevalence and morbidity associated with PPH in PD.

When examining these cases collectively, the findings suggest that the use of an abdominal belt in patients with PD and OH may help alleviate symptoms by increasing daytime BP. This aligns with the results of previous studies showing that external compression garments can relieve orthostatic symptoms by enhancing venous return and increasing central blood volume [15].

Our study also highlights the diurnal BP fluctuations in patients with PD. In clinical practice, patients often report experiencing daytime consciousness disturbances, and our findings specifically confirm that post-lunch BP decreases are particularly significant. Although the abdominal belt was effective in preventing severe hypotensive episodes, it did not completely eliminate postprandial hypotension. This is consistent with the results of previous studies showing that postprandial hypotension is less responsive to conventional treatments and may require a multifaceted approach, including dietary modifications, pharmacological interventions, and physical countermeasures [17,18].

We suggest that while the use of an abdominal belt may contribute to an increase in BP, it is unlikely to suppress fluctuations entirely. Variations in the extent and pattern of BP changes were observed among patients. Potential contributing factors to these differences include disease progression, severity of autonomic dysfunction, and body composition. Autonomic impairment in PD plays a critical role in BP dysregulation, underscoring the need for targeted interventions [14]. The importance of autonomic dysfunction in BP variability across neurodegenerative diseases is well-documented [3,8].

The use of abdominal belts was found to have a measurable effect in improving symptoms of OH. However, recent studies have also emphasized the impact of BP stabilization on non-motor symptoms, such as fatigue and cognitive function in PD. Maintaining a stable BP may contribute to improved cognitive function and reduced fatigue [19]. Additionally, recent research has indicated a potential link between effective OH management and overall quality of life in patients with PD [20].

This series has some limitations. First, its small sample size restricts the generalizability of the findings. Future research should focus on larger patient populations to validate these results. We also included only one control case, and the exclusion of confounding factors and potential placebo effects was not sufficient to determine the extent of improvement attributed solely to the abdominal belts. Indeed, the potential contribution of placebo effects is important considering the subjective nature of several reported outcomes. Additionally, this study only assessed short-term effects; further research is needed to evaluate long-term outcomes and patient adherence to abdominal belt use. Second, there is a lack of objective monitoring of belt usage in the reported cases. Although patients were instructed to wear the belt from 07:00 to 22:00, adherence was self-reported and may be subject to recall or reporting bias. Future studies incorporating prolonged ABPM could provide deeper insights into the sustained benefits of abdominal compression therapy. Third, as this was a case series of patients selected from a single institution, selection bias may have influenced the outcomes. Further research with larger, more diverse populations is needed to validate these findings and establish clearer patient selection criteria.

Despite these limitations, our study underscores the potential role of abdominal belts as an adjunct treatment for OH in PD. Given that current clinical guidelines do not explicitly recommend abdominal belts, our findings suggest that incorporating them into standard treatment strategies may be beneficial [17,18,20]. Future studies should incorporate validated symptom scales to assess clinical benefit, quantify actual device compliance, and define thresholds for clinically meaningful BP changes. In addition, effect size reporting and long-term follow-up data will be critical for evaluating therapeutic sustainability.

## Conclusions

This study indicates that the use of an abdominal belt in patients with PD and OH may help alleviate symptoms related to daytime hypotension by increasing BP. However, while the abdominal belt was found to contribute to increasing BP levels, it had limited impact on reducing fluctuations. Overall, these results could be a promising indication of the efficacy of non-pharmacological management methods in such cases.

Nevertheless, these findings should be considered preliminary, as they are based on a small case series without a control group. Furthermore, this symptomatic benefit was derived from patients’ self-reports, and no validated symptom assessment tools were employed in this study. Further research is required to develop standardized guidelines for the integration of abdominal belts into the treatment of OH in PD.
